# Diversity of Selected *Lupinus angustifolius* L. Genotypes at the Phenotypic and DNA Level with Respect to Microscopic Seed Coat Structure and Thickness

**DOI:** 10.1371/journal.pone.0102874

**Published:** 2014-08-13

**Authors:** Jon Clements, Renata Galek, Bartosz Kozak, Dariusz Jan Michalczyk, Agnieszka Iwona Piotrowicz-Cieślak, Ewa Sawicka-Sienkiewicz, Stanislaw Stawiński, Dariusz Zalewski

**Affiliations:** 1 Department of Genetics, Plant Breeding and Seed Production, Wroclaw University of Environmental and Life Sciences, Wroclaw, Lower Silesia, Poland; 2 Department of Plant Physiology and Biotechnology, University of Warmia and Mazury, Olsztyn, Warmian-Masurian, Poland; 3 Centre for Plant Genetics and Breeding, University of Western Australia, Crawley, Western Australia, Australia; 4 Plant Breeding Station, “HR Smolice Sp. z o. o. Grupa IHAR” Przebedowo, Greater Poland, Poland; USDA- ARS, United States of America

## Abstract

The paper investigates seed coat characteristics (as a percentage of overall seed diameter) in *Lupinus angustifolius* L., a potential forage crop. In the study ten *L. angustifolius* genotypes, including three Polish cultivars, two Australian cultivars, three mutants originated from cv. ‘Emir’, and one Belarusian and one Australian breeding line were evaluated. The highest seed coat percentage was recorded in cultivars ‘Sonet’ and ‘Emir’. The lowest seed coat thickness percentage (below 20%) was noted for breeding lines 11257-19, LAG24 and cultivar ‘Zeus’ (17.87%, 18.91% 19.60%, respectively). Despite having low seed weight, the Australian line no. 11257-19 was characterized by a desirable proportion of seed coat to the weight of seeds. In general, estimation of the correlation coefficient indicated a tendency that larger seeds had thinner coats. Scanning Electron Microscopy images showed low variation of seed coat sculpture and the top of seeds covered with a cuticle. Most of the studied genotypes were characterized by a cristatepapillate seed coat surface, formed by elongated polygonal cells. Only breeding line no. 11267-19 had a different shape of the cells building the surface layer of the coat. In order to illustrate genetic diversity among the genotypes tested, 24 ISSR primers were used. They generated a total of 161 polymorphic amplification products in 10 evaluated narrow-leaved lupin genotypes.

## Introduction

Besides the yellow lupin *Lupinus luteus*, the narrow-leaved lupin (NLL) *L. angustifolius* is the main lupin species currently cultivated in Poland and world-wide. As compared with the yellow or white lupine *Lupinus albus*, the narrow-leaved lupin has grown in importance during the recent years due to its improved yield, lowered seed alkaloid level and acquisition through selection of forms characterized by field tolerance to anthracnose [1], [2]. Amongst the cultivated lupin species, this particular lupin is additionally distinguishable by the shortest growing season. Since the 1980s the narrow-leaved lupin has been playing the most important role in Australia, where it is mainly cultivated as a crop in rotation with cereals or used as forage for feeding sheep, pigs and poultry. The low alkaloid content, introduced into the cultivars and maintained at the same level, allowed to develop a large-scale export of this crop from Australia, especially to Asiatic markets [3]. The possibility to treat lupins as alternative source of protein is more and more frequently taken into consideration, for they could replace the transgenic soybean in animal foodstuff. Moreover, thanks to mixed sowing of different lupin species with cereal plants, especially with the spring triticale, and their use as intercropping or green forage, lupins are increasingly popular in modern agriculture [2], [4]. While already highly valuable, cultivated lupins have some characters that should be improved through breeding.

Research on lupins and also that on other leguminous plants, such as the pea, bean or soybean, involves studies which are aimed at acquisition of thin-walled pods and the thinnest possible seed coat [5], [6], [7]. A proportionally lower share of walls in a pod substantially facilitates the transport of assimilation substances to the seeds. The narrow-leaved lupin is characterized by the proportion of pod walls ranging from 32% to 35%, whereas in the Andean lupin this value reaches 29–47%. Through induction of *Lupinus angustifolius* mutations from cv. ‘Emir’ and subsequent selection, it was possible to obtain forms of 24–30% proportion of pod walls in a pod [8]. This value recorded for the pea approaches 13%, reaching 24% in the bean [6]. The proportion of seed coat in the seeds of the narrow-leaved lupin is about 24%, whereas in the soybean it is only 7%, and in the pea – 9%. Seeds deprived of their coat are characterized by better digestibility, which is an important feature for feeding monogastric animals [5], [6]. Among the material comprised within lupin collections [8], seed coat precentages as low as 19–20% have been found. The formation process, structure and morphology of the seed coat in lupins have been extensively analyzed and the resulting diagnostic differentiation allowed the genus *Lupinus* to be divided into two sections, smooth-seeded and rough-seeded, confirmed also by scanning electron microscope (SEM) studies [9], [10], [11].

The breeding process of lupins requires genetic analysis and application of the new biotechnological methods to be more intensified and accelerated. The knowledge about the lupine genome organization and genetic background of important genes is still limited [12], [13], [14], [15]. While Australian lupin plant breeding has selected for seed coat thickness, so far Polish lupin plant breeding, for a thin seed coat has been not conducted and the relationship between phenotypic and genotypic diversity has not been studied. The aim of this study was to evaluate the variability of selected genotypes of lupin in terms of the SEM structure of the seed coat and its thickness, thousand seed weight and genetic diversity at the DNA level based on the inter-simple sequence repeat (ISSR) markers.

## Materials and Methods

### Phenotypic evaluation of seed coat thickness

#### Material

For the study, seeds of ten narrow-leaved lupin genotypes were chosen, which included three Polish and two Australian cultivars, three mutants of cultivar ‘Emir’, an Australian and a Belarusian breeding line (Table 1).

**Table 1:** Weight of 1000 seed and seed coat percentage in analysed genotypes of *Lupinus angustifolius* L.

Genotype	Weight of 1000 seed (g)									Seed coat (%)								
	Mean									Mean								
Years	2006–2007		SEM	2006		SEM	2007		SEM	2006–2007		SEM	2006		SEM	2007		SEM
AU-11257-19	148.6	DE	2.41	145.3	C	1.86	151.9	D	1.82	17.9	D	0.39	18.7	D	0.17	17.0	C	0.08
PL-41H	157.8	D	3.95	166.3	B	1.39	149.3	D	1.95	20.9	CD	0.56	21.6	BC	1.01	20.2	AB	0.23
PL-44H	142.9	E	3.91	134.8	D	1.01	151.0	D	3.11	21.7	BCD	0.67	23.1	AB	0.20	20.2	AB	0.09
PL-53H	143.0	E	5.86	130.9	D	5.03	155.1	D	0.27	22.7	AB	0.17	25.5	A	2.43	19.8	AB	0.10
PL-Emir	142.1	E	6.21	128.5	D	2.73	155.7	D	1.05	22.7	AB	0.12	25,0	A	1.24	20.5	AB	0.18
BY-LAG24	194.3	B	7.75	195.1	A	3.06	193.4	B	5.71	18.9	EF	0.11	18.9	D	1.39	18.9	BC	0.03
PL-Sonet	143.9	E	4.58	128.1	D	6.86	159.7	D	1.91	23.4	A	0.83	25.3	A	0.46	21.5	A	0.19
AU-Walup	175.7	C	6.07	167.8	B	3.53	183.6	C	3.85	21.6	BC	0.70	23.3	AB	0.46	19.8	AB	0.10
AU-Yorrel	206.3	A	2.90	194.2	A	3.80	218.5	A	4.79	20.9	CD	0.45	22.4	BC	0.43	19.4	ABC	0.10
PL-Zeus	174.6	C	2.37	168.8	B	2.69	180.4	C	3.32	19.6	DE	0.16	20.5	CD	0.31	18.7	BC	0.20
Mean	162.9			156.0			169.9			21.0			22.4			19.6		

Means for each year based of three replications ± standard errors.

A,B,C …-uniform groups based on the Duncan test.

AU-originated from Australia.

PL-originated from Poland.

BY - originated from Belarus.

SEM – standard error of the means.

Experiments with selected genotypes of lupin were conducted in two consecutive years, 2006 and 2007, according to the method of randomized block design in three replications. For each genotype, samples of pods with seeds were randomly collected (30 pods in each year, in each replication) at the stage of physiological maturity. The seeds were removed from the pods, and then from each sample ten healthy seeds were selected randomly and weighed. The 1000-seed weight was calculated. Evaluation of the seed coat proportion was conducted according to Clements et al. [6].

Seeds were hulled for each replicate. Ten healthy seeds were randomly selected and weighed. They were then placed in Petri dishes in a small amount of water at 4°C for 24 h. From swollen seeds the seed coat was removed so that the cotyledons and embryo were not damaged. Prepared samples of the seed coats, seed and cotyledons with embryo were dried separately for 48 h at 105°C, and then dried to dry matter. The dried seed coats and embryos with cotyledons were placed in a desiccator to cool and then weighed separately.

The seed coat proportion was reported as a percentage relative to the dry weight of seed coats of whole seeds.

#### Statistical analysis

A variance analysis was carried out in order to verify the hypothesis of lack of difference in the means of the genotypes studied with respect to the 1000-seed weight and the seed coat proportion. The analysis of variance (ANOVA) model for the individual data is as follows:
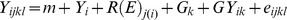
where Y_ijkl_ is the weight of 1000 seeds and seed coat of the levels k of genotype (G), i of year (Y) factors, R(E)_j(i)_ is the effect of the j block within the i years, GY_ik_ is the interaction effect of the k genotypes with the i years, e_ijkl_ is the random error component. In Table 2 are therefore only relevant effects.

**Table 2:** Mean squares from variance analysis for 1000-seed weight and seed coat percentage.

Source of variability	Degrees of freedom	Weight of 1000 seed (g)	Seed coat (%)
Genotypes (G)	9	3325.7**	0.0019**
Years (Y)	1	2891.3**	0.0121**
Interaction (G×Y)	9	328.8**	0.0004*
Error	40	34.9	0.0002

*significance at the level of α = 0.05.

**significance at the level of α = 0.01.

The mean values of these two traits were grouped using the Duncan test. To show the relationship between the traits under analysis, a correlation coefficient and linear regression were calculated.

#### Scanning electron microscopy

**Figure 1:**
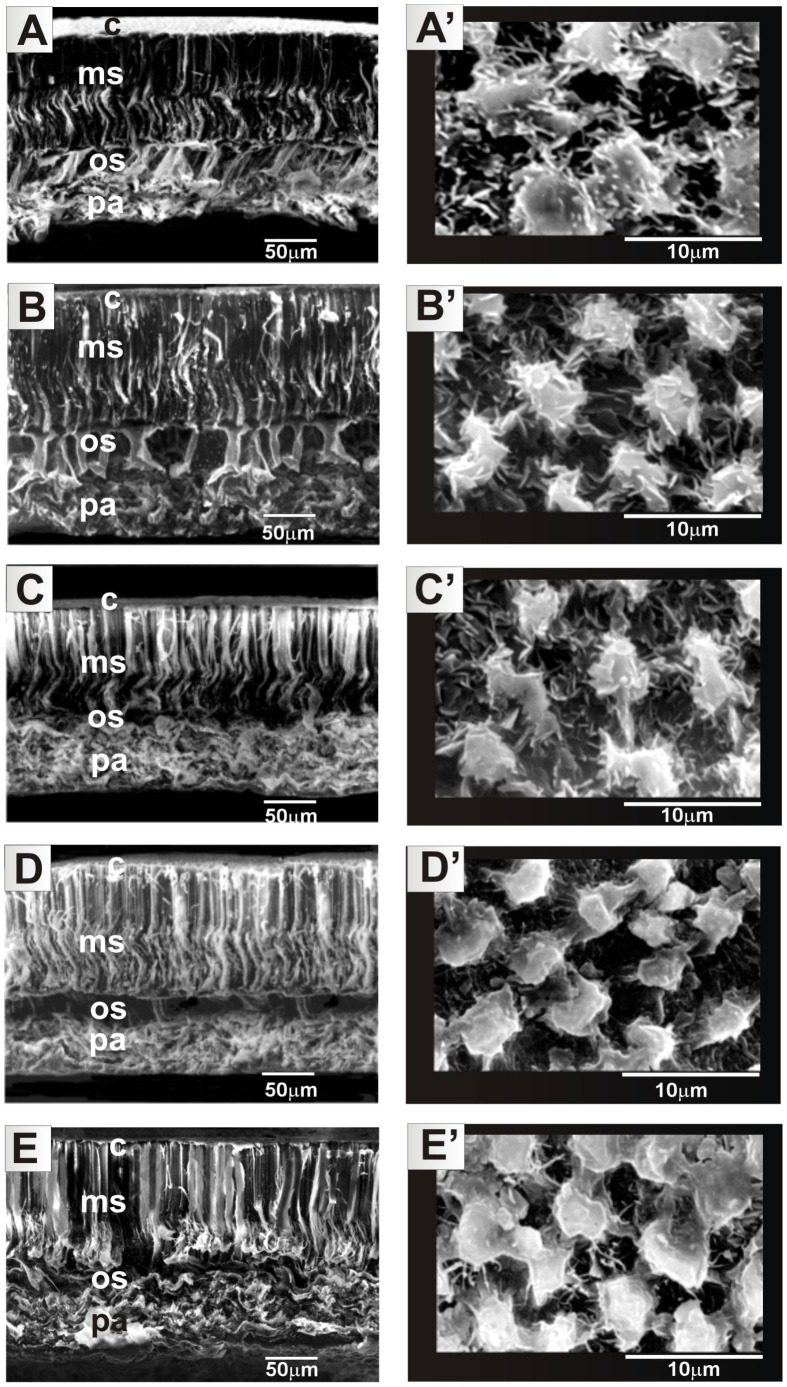
*Lupinus angustifolius* seed sculpture in the mutant lines PL-41H (A, A′), PL-44H (B, B′), PL-53H (C, C′), PL-‘Emir’ (D, D′), PL-‘Sonet’ (E, E′). SEM images of seed surface (A′, B′, C′, D′, E′ – general view of seeds in inserts) and cross-section of the seed coat (A, B, C, D, E). C – cuticle; MS – macrosclereids layer; OS – osteosclereids layer; PA – parenchymal cells.

**Figure 2:**
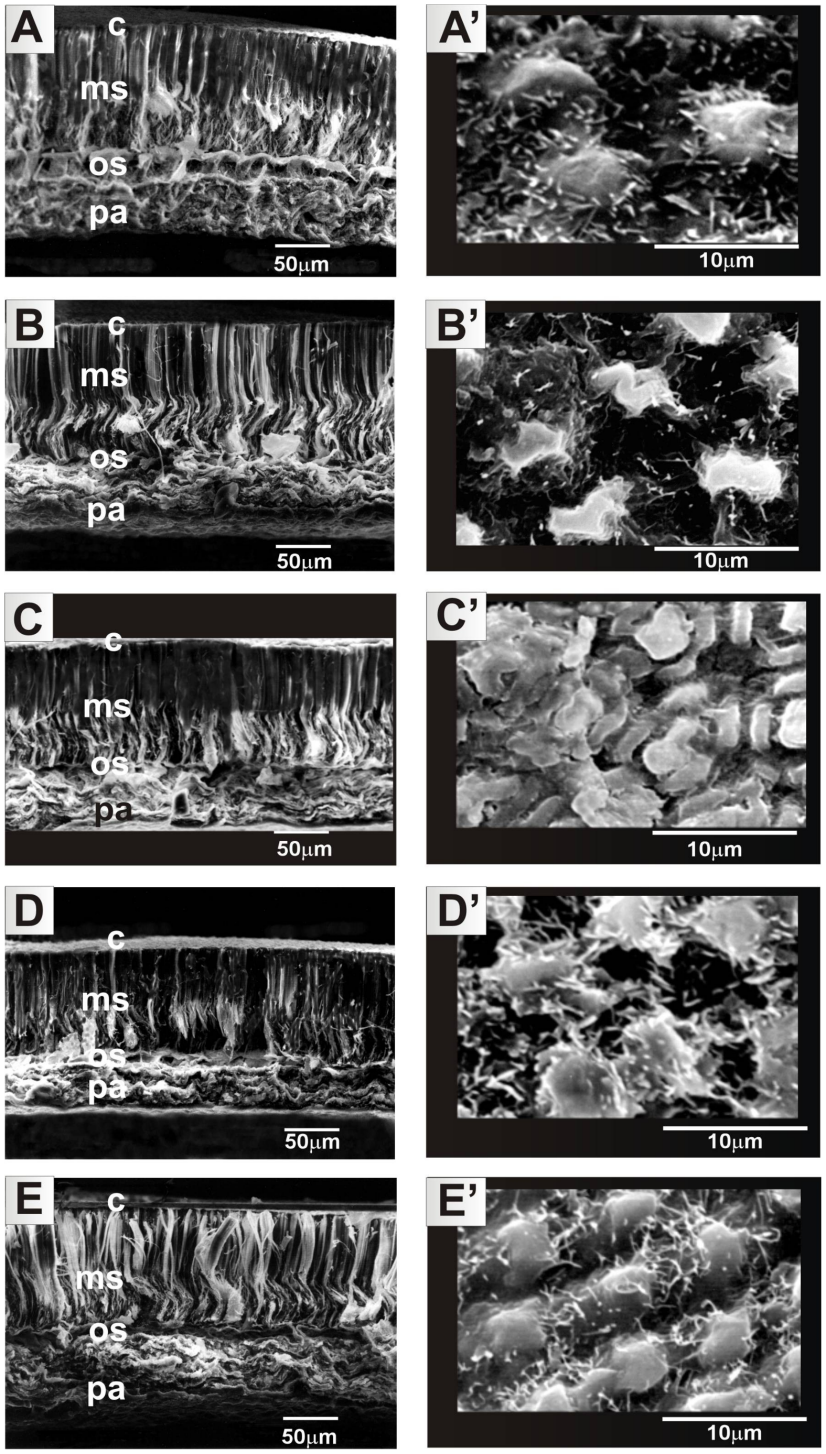
*Lupinus angustifolius* seed sculpture in PL-Zeus (A, A′), BY-LAG24 (B, B′), AU-11267-19 (C, C′), AU-Walup (D, D′), AU-Yorrel (E, E′). SEM images of the seed surface (A′, B′, C′, D′ E′, – general view of seeds in inserts) and cross-section of the seed coat (A, B, C, D, E). C – cuticle; MS – macrosclereids layer; OS – osteosclereids layer; PA – parenchymal cells.

The seed surface and cross-sections were observed by SEM in four replications (seeds).Specimens were coated with gold using a JEOL JFC 1200 ion coater and observed in a JEOL JSM – 5310 LV scanning electron microscope under 20 kV. In Figures 1–2, the surface layer of the cuticle (C), the layer of palisade cells (MS – macrosclereids), the supportive layer (OS – osteosclereids) and the layer of parenchymal cells (PA) are distinguishable.

### DNA polymorphism characteristics

#### Molecular analysis

Genomic DNA was extracted from young leaves of each of 10 collection lines, using the cetyltrimethyl ammonium bromide (CTAB) method of Doyle & Doyle [16] with minor modification. The optimum annealing temperature (Ta) of each ISSR primer was determined by performing a PCR reaction in one lupin genotype using a gradient thermal cycler. Initially 100 ISSR primers were tested. On the basis of the maximum number of reproducible and distinctly scorable polymorphic bands, a set of 24 ISSR primers from the University of British Columbia list (#UBC 9) was used (Table 3). Amplification of DNA was performed in a 15 µl reaction mixture according to Zietkiewicz et al. [17], using a 2×PCR mixture that contained Taq polymerase 0.1 U/ul, dNTP mix 2 mM, MgCl_2_ 4 mM (AA Biotechnology, Poland), 0.53 µM primer and 45 ng of DNA using a Biometra thermal cycler for 40 cycles. After initial denaturation for 5 min at 94°C, each cycle was composed of 30-sec denaturation at 94°C, 30-s annealing at 43–53°C depending on the primer (Table 3), 45-s extension at 72°C with a final extension for 7 min at 72°C at the end of 40 cycles. The amplified products were analyzed with the application of the QIAxcel (Qiagen, Germany) capillary electrophoresis system with the QIAxcel DNA screening Kit (Qiagen, Germany) and the AM420 method. The QX Alignment Marker 15 bp/3 kbp (Qiagen, Germany) and QX DNA Size Marker 100 bp–2.5 kbp (Qiagen, Germany) were used in the analysis. Molecular analyses were performed for two genomic replications.

**Table 3:** Nucleotide sequence and annealing temperatures of primers generating polymorphic ISSR products in the analysed genotypes.

Primer number in UBC #9	Nukleotide sequence (5′-3′)*	Annealing temperatures (°C)	Primer number in UBC #9	Nukleotide sequence (5′-3′)*	Annealing temperatures (°C)
807	AGA GAG AGA GAG AGA GT	53	841	GAG AGA GAG AGA GAG AYC	43
808	AGA GAG AGA GAG AGA GC	53	868	GAA GAA GAA GAA GAA GAA	43
810	GAG AGA GAG AGA GAG AT	53	876	GAT AGA TAG ACA GAC A	43
825	ACA CAC ACA CAC ACA CT	53	886	VDV CTC TCT CTC TCT CT	43
855	ACA CAC ACA CAC ACA CYT	53	811	GAG AGA GAG AGA GAG AC	48
889	DBD ACA CAC ACA CAC AC	53	817	CAC ACA CAC ACA CAC AA	48
890	VHV GTG TGT GTG TGT GT	53	818	CAC ACA CAC ACA CAC AG	48
881	GGG TGG GGT GGG GTG	53	827	ACA CAC ACA CAC ACA CG	48
814	CTC TCT CTC TCT CTC TA	43	836	AGA GAG AGA GAG AGA GYA	48
815	CTC TCT CTC TCT CTC TG	43	842	GAG AGA GAG AGA GAG AYG	48
820	GTG TGT GTG TGT GTG TC	43	864	ATG ATG ATG ATG ATG ATG	48
828	TGT GTG TGT GTG TGT GA	43	867	GGC GGC GGC GGC GGC GGC	48

*R purine, Y pyrimidine; N any nucleotide; B indicates C, G or T; D as A, G, or T; H as A, C, or T; and V as A, C, or G.

#### Statistical analysis

For grouping objects based on genetic distances cluster analysis (CA) was performed using the unweighted pair-group method with arithmetic mean (UPGMA). Each fragment that had been amplified was treated as a unit character and scored in terms of a binary code (1/0 = +/−). Data were entered in the Qiaxcel Screening Gel Software (Qiagen, Germany) and in a Microsoft Excel spreadsheet to create a binary matrix. The basic genetic diversity parameters were calculated: the polymorphism of amplification products (P – %), the mean number of observed alleles (na), the mean Nei gene diversity index (Dh) and PIC (Polymorphism Information Content), using the PowerMarker Software [18]. A matrix of the Nei genetic identity (Is) and genetic distance (Ds = 1−Is) was calculated according to the formula Is = 2Nij/(Ni+Nj), where Nij is the number of bands present in both genotypes i and j, Ni is the number of bands present in genotype i, and Nj is the number of bands present in genotype j [19]. For calculation of Is and Ds the NTsys_pc2.2 software was used [20]. The mean number of effective alleles (ne) and the Shannon index (I) were evaluated by the POPGEN software [21].

The dendrogram was developed with the Treecon ver 1.34b software [22] based on the Nei genetic diversity matrix and a UPGMA cluster analysis [23].

## Results and Discussion

### Phenotypic characteristics of seed coat thickness

The performed variance analysis revealed significant diversification of the genotypes studied with regard to the proportion of the seed coat to the seed size. What is more, the climatic conditions were found to significantly affect the analyzed traits (Table 2). The thickest seed coat, ranging from 23.4% to 22.7%, was characteristic of two Polish cultivars, namely ‘Sonet’ and ‘Emir’, and the mutant line 53H (Table 1). Based on the Duncan test, these genotypes were classified to one uniform group; simultaneously, these forms were distinguishable by the smallest seeds of the whole group under analysis. In the research pursued in 2003 and 2004 [24], it was also cultivar ‘Emir’ that – amongst the 34 genotypes studied – displayed the least favourable proportion of seed coat, reaching as much as 27%. The lowest percentage of seed coat, i.e. below 20%, was noted for the Australian line 11257-19, Belarusian breeding line LAG24 and Polish cultivar ‘Zeus’ (Table 1). In 2003–2004 also LAG24 and ‘Zeus’ represented the five best of the 34 forms under analysis (19–20%). The Australian line 11257-19 – in spite of having tiny seeds – was distinguishable by an advantageous proportion of seed coat, which is of great importance from the breeding perspective. The Australian cultivars ‘Yorrel’ and ‘Walup’ had approximately the same seed coat proportion (21–22%), also confirmed by their classification based on the Duncan test, which placed them within one uniform group. The seeds produced by these cultivars, and especially ones of ‘Yorrel’, belonged to the largest (Table 1). In half of the studied forms the weight of 1000 seeds equalled 144 g on average; these genotypes constituted group “E”. All these genotypes, except for line 11257-19, were characterized by an 22.6% average proportion of seed coat. A comparison between the seed size obtained in the experiments carried out in 2003–2004 [24] and that for 2006–2007 shows tendency to develop large seeds by the same genotypes, in spite of significant interaction between “G×Y” (Table 2). In both cycles of experiments, the cultivar ‘Zeus’ and breeding line LAG24 were characterized by the largest seeds (174 and 194 g respectively).

**Figure 3:**
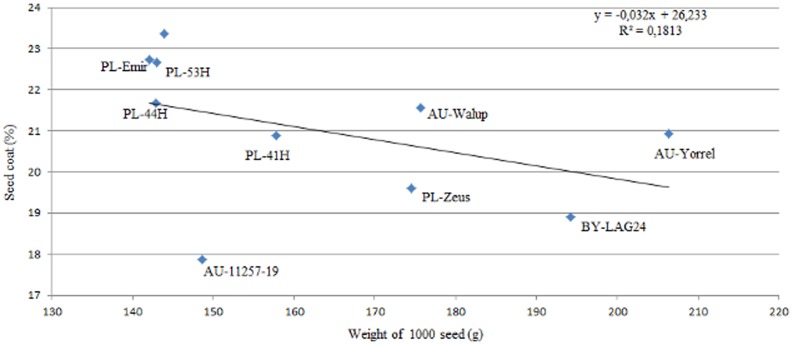
The linear regression between weight of 1000 seeds and seed coat for analysed genotypes *Lupinus angustifolius*.

An analysis of dependence between the size of seeds and the percentage of seed coat based on the correlation coefficient (r = −0.43, p>0.05) revealed a negative correlation (Figure 3), similarly as it was ascertained in the research by Sawicka-Sienkiewicz et al. [24]. The correlation coefficient indicates the tendency that genotypes of larger seeds have a thinner seed coat (Figure 3). Such a relationship was not detected in the Australian line 11257-19, which points to the possibility that genotypes of fairly small seeds and simultaneously with a thinner seed coat. The lack of linear correlation between analysed traits gives breeders the opportunity to select genotypes with medium seed weight - and low seed coat proportion. Genetic-breeding investigations should also take into consideration another significant factor, i.e. presence of the genotype×year interaction, which affects the expression of the analyzed traits depending on the fluctuations in climatic conditions within a given year [6], [7], [24].

The formation, structure and morphology of the seed coat in legumes have been widely analysed (Newell & Hymowitz 1978; Gunn 1981; Trivedi et al. 1987; Buth & Narayan 1987; Harris 1987; Manningand Van Staden 1987, quoted after Planchuelo and Perisse [10] Michalczyk et al. [11] by different groups of researchers. Almost all anatomical studies agree that the following layers can be distinguished in the transverse cross-section of the legume seed: (1) the external non-cellular layer or cuticle that covers the whole seed except the hilum; (2) the palisade layer with Malpighian or macrosclereid cells, which is the osteosclereid layer; (3) the crushed parenchyma cells; and (4) the remaining endosperm layer. Due to the variation in the thickness of the seed coat in the tested forms of lupin characterized by high and low weight of a thousand seeds, we carried out observations of the microscopic structure of the seed coat.

In the lupin genotypes analysed the seed coat was composed of the following layers, depicted in Figures 1 and 2: cuticule, macrosclereid layer, osteosclereid layer, and parenchymal cells. The seed coat thickness, visible in the photographs taken by SEM, in the particular cultivars and breeding lines of the NLL ranged from 170 µm (41H, 53H, ‘Zeus’, LAG24, 11257-19 and ‘Walup’) to 200 µm (44H, ‘Emir‘, ‘Sonet’, ‘Yorrel’). The macrosclereid layer was the thickest (100 µm) in all seeds, while the layer of osteosclereid was the thinnest (≥30 µm). Figures 1 and 2 (A′–E′) show that in most of the investigated genotypes the seed coat surface was cristate-papillate, formed by elongated polygonal cells, as described by Perrisé and Planchuelo [9]. It was only line 11267-19 that was characterized by a different shape of the cells building the surface layer of the coat; most of them took a cuboidal shape. The shape of cells which build the surface layer of the seed coat in lupins is species-specific and can be treated as a diagnostic character in the taxonomy of this group [10].

### ISSR polymorphism and basis genetic diversity parameters

**Figure 4:**
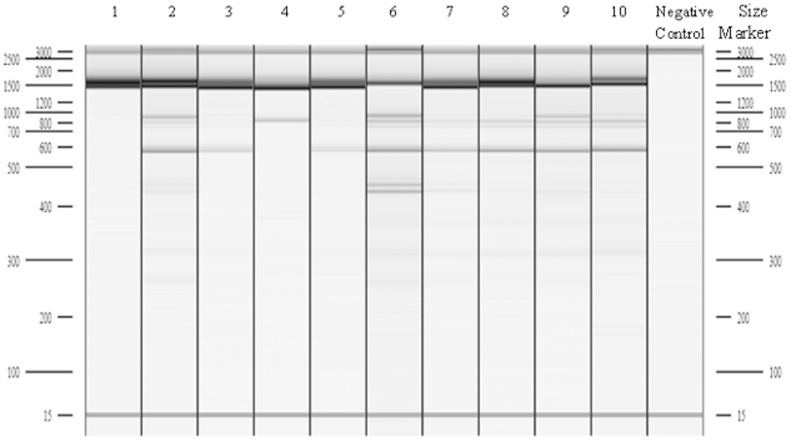
Products of PCR-ISSR amplification after using of UBC 815 for analysed genotypes *Lupinus angustifolius*: 1 – AU-11257-19/1, 2 – PL-Sonet, 3 – PL-53H, 4 – AU-Walup, 5 – PL-44H, 6 – AU-Yorrel, 7 – PL-41H, 8 – PL-Zeus, 9 – BY-LAG24, 10 – PL-Emir.

The 24 ISSR primers tested generated a total of 249 amplification products, of which 33% were monomorphic (Table 4). The total genotyping error rate of all ISSR markers used in this study was 4.6%. The number of products amplified using single primers ranged from 4 to 20 with an average of 10.4 products per primer. The size range of the analysed ISSR products was from 172 (for primer 807) to 2500 (for primer 881) bp. The average degree of homology between genotypes for each primer reached 69.2% (Table 4). The lowest degree of homology was - for primer ISSR 815 (54%) (Table 4, Figure 4) and the highest – for primer ISSR 855 (85%). The polymorphism of all amplification products (P) was 64.66% for the genotypes investigated. The mean number of observed alleles (na) per locus reached 1.65. The Nei gene diversity index equalled 0.25 (Dh) and the Shannon index was 0.36 (I). The mean number of effective alleles (ne) per locus was 1.65 in all genotypes.

**Table 4:** Characteristics of number amplifications products, degree of homology between the analysed genotypes and PIC values for ISSR primers.

Primer	Number of amplification products				Degree of homology of genotypes [%]		PIC	
	Total	Monomorphic	Polymorphic		Mean	Range	Mean	Range
807	13	4	9		66.2	10–100	0.194	0–0.375
808	15	6	9		72	30–100	0.176	0–0.375
810	7	3	4		75.7	30–100	0.194	0–0.365
825	4	2	2		77.5	50–100	0.185	0–0.375
855	7	5	2		88.6	40–100	0.091	0–0.365
889	4	2	2		75	40–100	0.182	0–0.365
890	11	7	4		73.6	20–100	0.112	0–0.365
881	19	6	13		70	20–100	0.220	0–0.375
814	9	1	8		61.1	30–100	0.294	0–0.375
815	10	1	9		54	10–100	0.267	0–0.375
820	5	2	3		70	20–100	0.183	0–0.375
828	15	6	9		71.3	10–100	0.193	0–0.375
841	8	3	5		58.8	10–100	0.144	0–0.332
868	12	4	8		65.8	10–100	0.189	0–0.375
876	10	7	3		84	40–100	0.109	0–0.365
886	20	7	13		65.6	10–100	0.208	0–0.365
811	6	3	3		85	50–100	0.145	0–0.375
817	9	5	4		71.1	10–100	0.130	0–0.375
818	6	1	5		55	20–100	0.293	0–0.375
827	11	4	7		65.4	10–100	0.157	0–0.365
836	11	4	7		78.2	20–100	0.163	0–0.332
842	17	0	17		57	10–90	0.300	0.164–0.375
864	12	5	7		63.3	10–100	0.147	0–0.375
867	8	0	8		56.25	30–90	0.331	0.332–0.375
Sum	249	88	161	Mean:	69.2			

### Cluster analysis

**Figure 5:**
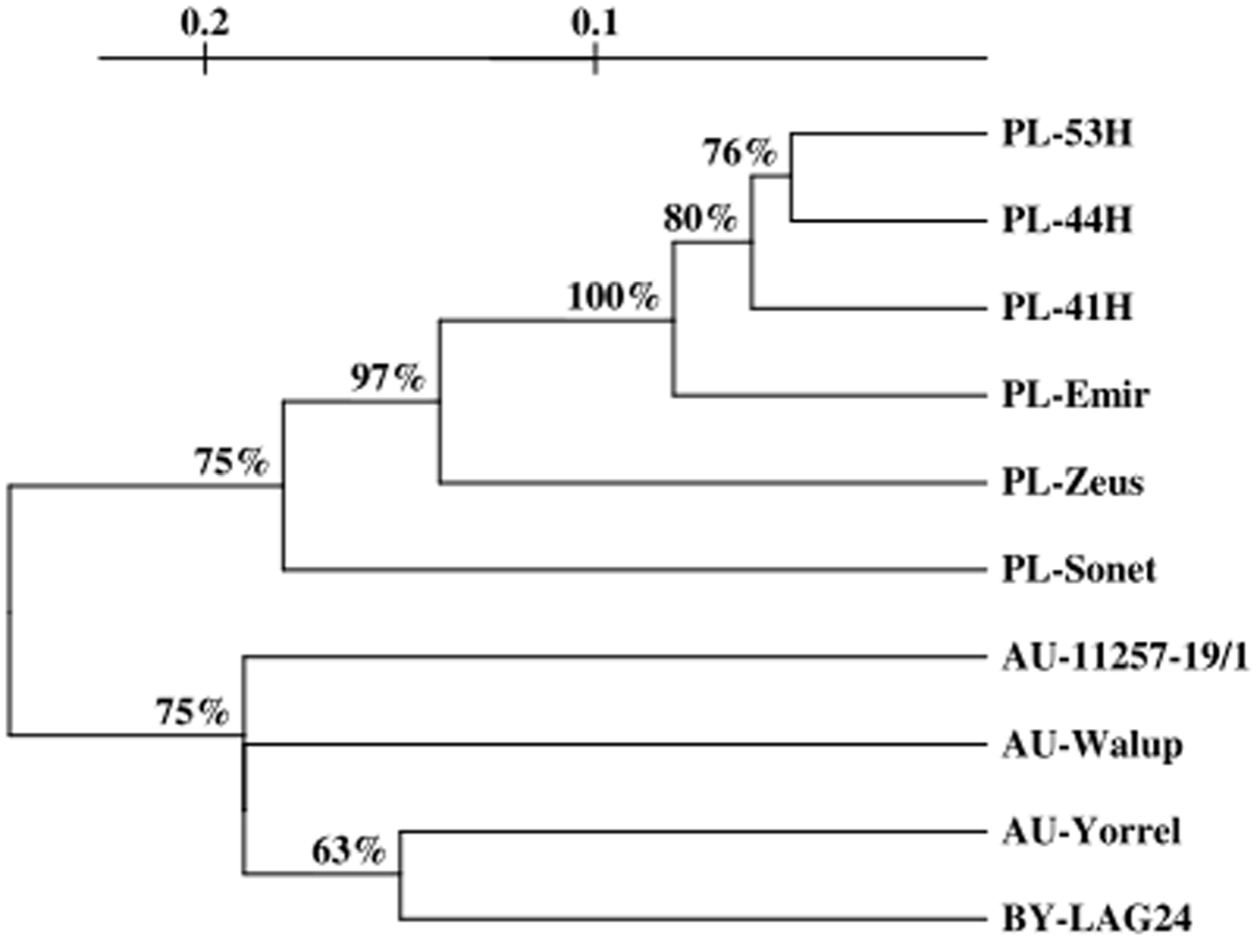
Derndrogram of 10 *Lupinus angustifolius* genotypes based on ISSR data using the Nei genetic distance matrix of similarity and UPGMA clustering method.

The Nei genetic identity index ranged from 0.050 (between 44H and 53H) to 0.311 (between cvs ‘Zeus’ and ‘Yorrel’) (Table 5). The genetic similarity between *L. angustifolius* genotypes fell within the range of 0.711 to 0.950 and the mean value was 0.798. The genetic similarity between *L. angustifolius* genotypes is shown in the UPGMA tree (Figure 5). Two main clusters were observed. The lupin genotypes originating from Poland, were placed in the first cluster, whereas the genotypes originating from Australia and Belarusian breeding line made the second cluster. A branch of 75 bootstrap replicates separated six genotypes from Poland from the second cluster and a branch of 75 bootstrap replicates separated the second cluster from the others. The first cluster divided into two main subclusters – one of them contained only cultivar ‘Sonet’ and the other formed further two subclusters. One subgroup was represented by ‘Zeus’ and the other divided into further two clusters – one of them represented by cv ‘Emir’ and the other by its mutants (41H, 44H, and 53H). A low level of genetic polymorphism between mutants and cv ‘Emir’ was observed. The genetic distances values were below 0.1, with the highest value of this coefficient noted for cvs ‘Zeus’ and ‘Yorrel’. The second main cluster consisted of three subclusters -that were represented only by the Australian breeding line 11257-19. In the Australian cultivar ‘Walup’ and very similar Australian cultivar ‘Yorrel’, and also Belarusian breeding line LAG24, the genetic distances ranged from 0.146 (between ‘Yorrel’ and LAG24) to 0.216 (between LAG24 and Australian breading line 11257-19) (Figure 5, Table 5).

**Table 5:** Genetic identity (Is - below the diagram) and genetic distance (Ds - above the diagonal) statistics for the analysed genotypes of *L. angustifolius*.

	AU-11257-19	PL-Sonet	PL-53H	AU-Walup	PL-44H	AU-Yorrel	PL-41H	PL-Zeus	BY-LAG24	PL-Emir
AU-11257-19/1	***	0.198	0.235	0.202	0.256	0.157	0.231	0.259	0.216	0.247
PL-Sonet	0.802	***	0.171	0.200	0.185	0.258	0.177	0.199	0.232	0.176
PL-53H	0.765	0.829	***	0.233	0.050	0.290	0.064	0.140	0.282	0.091
AU-Walup	0.798	0.800	0.767	***	0.254	0.196	0.252	0.263	0.175	0.233
PL-44H	0.744	0.815	0.950	0.746	***	0.289	0.065	0.149	0.287	0.070
AU-Yorrel	0.843	0.742	0.710	0.804	0.711	***	0.269	0.311	0.146	0.261
PL-41H	0.769	0.823	0.936	0.748	0.935	0.731	***	0.135	0.261	0.085
PL-Zeus	0.741	0.801	0.860	0.737	0.851	0.689	0.865	***	0.254	0.151
BY-LAG24	0.784	0.768	0.718	0.825	0.713	0.854	0.739	0.746	***	0.253
PL-Emir	0.753	0.824	0.909	0.767	0.930	0.739	0.915	0.849	0.747	***

Also, available literature provides sample information on the use of molecular markers, including ISSR markers, for evaluating genetic diversity in the genus *Lupinus* [25], [26], [27], [28].

Yorgancilar et al. [25] obtained 76 polymorphic bands from 11 selected ISSR primers, of which 98.6% displayed polymorphism among the 20 genotypes belonging to three species: *L. angustifolius*, *L. luteus* and *L. albus*. The genetic distances calculated with the Jaccard coefficient ranged from 0.37 to 0.96. Range of diversity obtained in this was lower. Yorgancilar et al. [25] characterized in theirs investigations representatives of three above mentioned lupin species, in our cases only one species was studied.

Talhinhas et al. [27] used AFLP (*amplified fragment length polymorphism*) and ISSR markers to evaluate the genetic diversity among 88 genotypes of *L. angustifolius*. Molecular markers grouped modern cultivars as sub-clusters within the wider diversity of wild germplasm. The three ISSR primers considered for analysis gave a total of 25 bands (8.3 bands per primer and 5.6 bands per primer and accession), producing a total of 6 monomorphic bands (24.0% of the total). The similarity between accessions ranged from a minimum of 0.5185 to a maximum of 0.9767, the average amounting to 0.7681. In our studies mean value of bands per primer was higher, similar as percentage of monomorphic products. These differences could resulted from higher number of primers used in our experiment. Talhinhas et al. [27] obtained higher diversity, which seems to be connected with kind of tested material **-** consisted domesticated and wild accession. We tested only cultivated genotypes.

The undertaken study was the first attempt to investigate the genetic diversity of *L. angustifolius* gathered in the collection at the Department of Genetics, Plant Breading and Seed Production, Wrocław University of Environmental and Life Sciences. A whole set of UBC ISSR primers were pre-tested. Finally, using a set of 24 primers, ten genotypes were tested. The high repeatability of ISSR markers [29], [30] was obtained mostly due to the use of longer primers (16–25 bp) and higher annealing temperatures (45–65°C) [31]. The optimum annealing temperatures (Ta) of ISSR primers used in this study were 43°C, 48°C and 53°C, and the primers' length ranged from 16 to 18 bp (Table 3). Each primer accounted for an average of 10.4 ISSR bands. Most of the primers were informative and 161 polymorphic bands were produced with them – on average 6.7 per primer (Table 4). Based on the results of ISSR markers, the genetic diversity of the main parameters was calculated: na, Dh, ne and Shannon (I). An analysis of the values of these parameters supports the conclusion that the genetic diversity of the analysed genotypes was not too high, but enabled 10 genotypes to be grouped depending on the origin – Polish, Belarusian or Australian. This was understandable, since their taxonomic affinity was similar, and the number of analysed cases small compared with other studies, not only for the genus *Lupinus*. ISSR markers are thought to be particularly useful for study of closely related individuals that exhibit low levels of polymorphism [17] and have been applied as a very useful alternative to fingerprinting and genetic analysis in the case of *L. angustifolius*. This set of primers could be used in further studies on the genetic diversity of *L. angustifolius* in the breeding materials. The outcome of such research would facilitate the selection of appropriate genotypes for production of hybrids with a thin seed coat and for development of a mapping population. Subsequent research efforts should focus on the identification of quantitative trait loci (QTL) responsible for this trait in order to increase the breeding intensity with the application of marker-assisted selection (MAS).

## Conclusions

1. Breeding lines no. 11257-19 and LAG24, mutant line no. 41H and cultivar ‘Zeus’ can be used in breeding programmes as parental forms, to enhance the values of the seed coat thickness in the segregating progenies or further generations. Through inducing mutations and subsequent selection, desirable genotypes with a low percentage of seed coat in seeds have been obtained.
2. Mutant line no. 41H, derived from the cultivar ‘Emir’, had a significantly thinner seed coat as compared with the initial cultivar.
3. Moreover, the shape of cells that build the surface layer of the seed coat in lupins is species-specific and can be used as a diagnostic character not only in the taxonomy of two sections: smooth-seeded and rough-seeded. The exeption is line 11267-19 which was characterized by different shape of the cells building the surface layer of the coat.
4. Molecular and phenotypic studies facilitate the selection of favourable genotypes for the production of hybrids with the thickness coat in *L. angustifolius* breeding, and for development of a mapping population. Subsequent research efforts should focus on the identification of molecular markers (MAS) correlated with the thickness of the seed coat.
